# The costs of electoral fraud: establishing the link between electoral integrity, winning an election, and satisfaction with democracy

**DOI:** 10.1080/17457289.2017.1310111

**Published:** 2017-04-10

**Authors:** Jessica Fortin-Rittberger, Philipp Harfst, Sarah C. Dingler

**Affiliations:** ^a^ Department of Political Science, University of Salzburg, Salzburg, Austria

## Abstract

Previous research has shown that voters’ perception of electoral fairness has an impact on their attitudes and behaviors. However, less research has attempted to link objective measurements of electoral integrity on voters’ attitudes about the democratic process. Drawing on data from the Comparative Study of Electoral Systems and the Quality of Elections Data, we investigate whether cross-national differences in electoral integrity have significant influences on citizens’ level of satisfaction with democracy. We hypothesize that higher levels of observed electoral fraud will have a negative impact on evaluations of the democratic process, and that this effect will be mediated by a respondent’s status as a winner or loser of an election. The article’s main finding is that high levels of electoral fraud are indeed linked to less satisfaction with democracy. However, we show that winning only matters in elections that are conducted in an impartial way. The moment elections start to display the telltale signs of manipulation and malpractice, winning and losing no longer have different effects on voter’s levels of satisfaction with democracy.

## Introduction

This article investigates the impact of electoral misconduct on citizens’ level of satisfaction with democracy (SWD). Using the broadest comparative research design to date, we analyze the effects of different levels of electoral misconduct on citizens’ perceptions of the quality of the democratic process. Electoral integrity is a central building block for the quality of democracy in general, and for electoral freedom and fairness in particular: our hypothesis is that voters will not consider elections suffering from obvious fraud as meaningful as electoral contests which do not show signs of such defects. These fraudulent practices, in turn, also influence voters’ level of satisfaction with the way democracy works. Although intuitive in theory, the impact of electoral fraud on voters’ attitudes has received scant comparative empirical attention to date. Understanding this relationship is of crucial importance considering that variations in SWD are consequential factors that undergird both the consolidation and the stability of democracies (Almond and Verba 1963; Lipset 1994; Norris 1999).

Our key finding is that high levels of electoral fraud are, indeed, linked to less satisfaction with democracy. The effects we reveal are observable both at the country-aggregate and individual levels. However, we also find that this relationship may not be straightforward, depending on the outcomes of the elections. In particular, winning an election might be more important to voters than the fairness and competitiveness in which elections are conducted. Thus, our analysis of the effects of voter’s proximity to the winning party allows us to uncover new and interesting insights into the interplay between fraud and winning elections. We find that winning only makes a difference on SWD in elections that are conducted in an impartial way. The moment elections start to display the telltale signs of manipulation and malpractice, winning and losing no longer exert different effects on voters’ attitudes.

To investigate these propositions, we offer empirical verifications in three steps. First, we present graphical evidence to demonstrate the effects of general “objective” measures of electoral fraud on individual perceptions of electoral fairness and satisfaction with democracy in the countries included in this study. In a second step, we use multi-level ordered logistic regressions to analyze the effects of individual and aggregate factors related to electoral fraud. Third, we examine in more detail one of the most influential factors driving satisfaction with democracy, the particular effect of a voter’s proximity to the winning party. We draw our cases from the countries included in the first two modules of the Comparative Study of Electoral Systems (CSES) (2003, 2007) data, covering the timespan between 1996 and 2004. Although mainly centered on advanced industrial democracies, our research design displays a broad geographic coverage as it also considers a number of new democracies in which competitive elections are held. Among the countries under investigation, some 35% of elections suffered from some form of fraud, ranging from relatively minor (e.g. technical issues in some polling stations) to major, that is, where election results are largely fabricated. This grants us sizable amounts of variance to investigate our hypotheses.

## The effects of electoral fraud on voters’ satisfaction with democracy

The literatures on SWD and diffuse system support advance a myriad of hypotheses to explain the distributions in these attitudes observed worldwide. Norris (2011) classifies this existing body of contributions in three categories. The first includes “demand-side” explanations, based on societal modernization (aspirational, educational and socioeconomic explanations) and attributes such as trust and social capital. The second group of explanations focuses on the role of the media, and how people learn about democracy. The third – on which this paper will concentrate – incorporates supply-side theories where the structural environment (constitutional arrangements), the policy performance, or the democratic process itself are considered as key drivers of attitudes associated with SWD. The arguments we set forth center on the fundamental feature of democratic processes: elections. There are two major determinants of SWD. First, several studies show that various indicators of economic performance are closely linked to country-level measures of SWD (e.g. Finkel, Muller, and Seligson 1987; McAllister 1999; Newton 2006). Second, SWD at the individual level is positively related to being on the winners’ side of an election (e.g. Anderson and Guillory 1997; Anderson and Tverdova 2001; Blais and Gélineau 2007; Bowler and Donovan 2002; Singh, Karakoç, and Blais 2012). Despite these advances, much remains unknown about the effects of the procedural quality of elections on both country-level and individual levels of SWD. Given the centrality of elections for the practice of democracy and how widespread and diverse electoral fraud is, we feel this aspect deserves empirical scrutiny.

A growing body of research shows that voters are sensitive to the competitive context of elections (e.g. Blais 2000; Franklin 2002, 2004; Jackman and Miller 1995; Karp and Banducci 2008; Norris 2004, 2014), and are affected by perceptions of procedural fairness (e.g. Dahlberg and Holmberg 2013; Gilley 2006; Magalhães 2016; Rothstein and Teorell 2008). With regard to the conduct of elections, Birch (2010) and Norris (2014) echo earlier contributions on Latin America and Africa (Alemika 2007; Bratton 1998; Bratton and van de Walle 1997; McCann and Domínguez 1998): Citizens who perceive elections to be fair are more likely to vote than those who have reservations about electoral procedures. However, most studies use citizens’ subjective perception of fairness; fewer empirical investigations to date have tested analogous hypotheses by means of indicators assessing electoral fraud from third-party observations, even if the two are closely related (Norris 2013). Examining the effects of different forms of electoral misconduct, Donno and Roussias (2012) find that pre-election misconduct directed at deterring the opposition has psychological effects on parties and voters, reducing the number of legislative parties. We go a step further in examining the effects of electoral misconduct on voters’ attitudes directly, rather than through an indirect chain of causality involving party system size: if there are psychological effects on voters, these should be observable at individual level.

Manifestations of electoral fraud affect individuals by constraining the electoral choices available and by adding a layer of arbitrariness between citizens’ preferences and their translation into seats. We posit that electoral fraud in general acts as an additional filter hindering citizens from shaping policy outcomes, and is likely to affect perceptions of accountability and responsiveness. Under conditions of electoral fraud, electoral figures can no longer be considered reliable expressions of the general will but become by-products of electoral manipulation (Schedler 2006, 8). Moreover, given that electoral fraud is said to undermine the functioning of democracy (Lehoucq 2003; Norris 2014), general manifestations of electoral malpractice should negatively impact citizens’ SWD.

We also hypothesize that the relationship between electoral integrity and levels of SWD is conditioned by the results of the elections for two reasons. First, there is abundant evidence that, at the individual level, being on the winning side of an election is influential for political attitudes, such as SWD. Winning in this context is mostly about voting for a party who forms the executive (Anderson et al. 2005; Henderson 2008; Singh, Karakoç, and Blais 2012). Grimes (2006) suggests that although perceptions of the fairness of decision-making processes impact citizens’ acceptance of its outcome, this effect is substantially smaller than the perceived utility of the outcome. In the case of elections, winning has probably the highest possible utility and should therefore matter more than the degree of procedural fairness attached to them. Anderson and Tverdova (2003), for instance, found that the negative linkage between SWD and corruption was weaker among those who had voted for the winning party in the previous election, suggesting that winning could be more important than fair and impartial procedures.

Other accounts of the determinants of political legitimacy, however, stress the importance of procedural factors. Gilley (2006) emphasizes the importance of general governance (including corruption control, rule of law and government effectiveness) in citizens’ assessment of the legitimacy of states. Also, Dahlberg and Holmberg (2013), building on Rothstein (2009) and Rothstein and Teorell (2008), conclude that impartial bureaucratic procedures matter more for SWD than electoral outcomes such as congruence between voters and representatives. Empirical evidence is therefore mixed when it comes to the relationship between SWD, the importance of fair procedures, and the question of winning or losing an election.

Moreover, some contributions have established that institutions condition the relationship between election victory and SWD: winners of an election in majoritarian systems are more satisfied with democracy than winners in consensual systems while the inverse relationship holds for those who lose. Anderson and Guillory (1997) hypothesize that these differences are rooted in unequal policy gains. Winning an election in majoritarian democracies usually amounts to supporting a single party government that is comparatively unhampered to implement its program. If voters place a high premium on policy gains, evaluations of democracy might hinge more on winning an election than on the manner how it is won. We therefore hypothesize that electoral fraud as a breach of procedural impartiality will influence SWD negatively, but that winning an election could change the shape of this relationship and have a positive effect on SWD – irrespective of the degree of electoral misconduct.

To summarize our argument, we expect that electoral fraud will influence citizens’ attitudes about democracy. Yet, we likewise expect that winning an election will also affect SWD irrespective of the quality of elections. Voters are interested in policy gains, regardless of whether these gains come at the price of electoral fraud. Therefore, while electoral fraud should negatively impact SWD in general, this relationship should weaken for those voters who vote for the winning party in fraudulent elections.

## Data and methodology

The individual-level data used to test the hypotheses are drawn from the first two modules of the CSES: we perform analyses on 48 elections over 29 countries.^1^ Given the strategic timing of CSES interviews, fielded on average within three months after an election, these data provide an optimal analytical context in which the most recent elections are equally salient to respondents. Our units of analyses are elections, including both legislative (lower house) and presidential elections. We also draw on aggregate-level data from a variety of sources detailed below to supplement the individual-level data from surveys.

The strengths of our approach are twofold. First, we use a combination of micro- and macro-level predictors to explain our dependent variables of interest and control for a variety of individual and election-level variables which previous research has found to influence SWD. Second, although most of the countries covered in the CSES data are advanced industrial democracies, where there has been little conspicuous electoral malpractice, there is a significant number of newly democratized countries from East Central Europe, the former Soviet Union, Latin America, and Asia. The advantage of this broad comparative sample is that our models have the potential to uncover patterns that are common to all groups of countries.

The empirical verification proceeds in three steps. First, we present graphical evidence to demonstrate the effects of electoral fraud on SWD in the countries included in this study, using general measures derived from third-party observations. In a second step, we use a series of multi-variate models to estimate the effects of this general concept on SWD. Third, we inspect the interaction between winning an election and the degree of fraud observed in this contest, and finally, whether electoral fraud affects voters the same way in established versus newer democracies. To accommodate our categorical response variable, the models were specified as multi-level ordered logistic regressions.

### Independent variables

#### Measuring electoral malpractice

Opening up the conceptual toolbox of electoral fraud, we find wide-ranging types of means through which candidates and parties can manipulate the electoral process during the campaign on election day as well as after the elections. The large number of competing conceptualizations of the phenomenon of electoral fraud (e.g. Birch 2008; Elklit and Reynolds 2005; Norris 2014; Schedler 2002) mirrors this diversity. Schedler (2002) points out that diverse forms of electoral fraud can occur at different stages of the electoral process and should be weighted equally. Drawing on this insight – and since we investigate the effects of fraud in both established and new democracies – we opted for a composite index using *Quality of Elections Data* (QED) (Kelley 2010, 2012; Kelley and Kolev 2010), combining a series of items, as proposed by Norris (2014). Beyond the overall election quality and the extent of problems, the composite measurement also takes into consideration multiple components of the election cycle, including the legal framework, campaign environment, electoral administrative capacity, election violence, and polling day fraud.^2^ As the items were standardized before aggregation was performed, the final measurement we use ranges from −0.4 (no problems) to 2.3 (major problems). In the remainder of the paper, we coin this index “magnitude of problems in elections.”^3^

#### Status of winner or loser of an election

Following an established literature (e.g. Anderson and Guillory 1997; Bernauer and Vatter 2012; Blais and Gélineau 2007), we operationalize whether citizens are on the winning or losing camp in each election by considering whether respondents have voted for the winning party, coalition party, or candidate. This operationalization is, however, performed at the cost of eliminating non-voters from the analyses.

### Dependent variable

To measure the level of SWD for individuals, we use a single item, asking respondents if they are satisfied with the way democracy works in their country from the CSES, ranging from 1 (very dissatisfied) to 4 (very satisfied), on an ordinal scale. By choosing this item, we subscribe to Linde and Ekman’s view (2003) that SWD is less an indicator of system legitimacy per se than an indicator of the support of the performance of the democratic regime. Using SWD thus fits our purpose since we are more interested in how democracy works in practice than in a system’s legitimacy. Despite these advantages, a growing literature urges researchers to proceed with caution when using a single indicator such as the ‘satisfaction with democracy’ measure (e.g. Linde and Ekman 2003; Wittrock 2010) as this indicator is sensitive to variations in the political environment. Since our research questions do not focus on respondents’ preferences for democracy as an ideal, but on the effects of the immediate political environment surrounding the elections, SWD remains the most appropriate item in our view.

### Control variables and alternative hypotheses

Our models comprise several control variables in order to rule out the effect of omitted, or rival, hypotheses on the dependent variable.

#### Individual factors

At the micro-level, we include the covariates of age, education level, income, a measurement of mobilization such as belonging to religious groups and membership in political parties, as well as an indicator of external efficacy (whether whom one votes for makes a difference).

#### Institutions

Since several studies found that institutional variables influence individual attitudes, such as SWD (e.g. Aarts and Thomassen 2008; Bernauer and Vatter 2012; Karp and Banducci 2008; Klingemann 1999; Lijphart 1999), the level of political engagement of citizens and efficacy (e.g. Blais and Carty 1990; Clarke and Acock 1989; Jackman and Miller 1995), we control for the potential cofounding effects of alternative constitutional designs on attitudes. Variables for the type of electoral rules (proportional vs. others) and type of executive (parliamentary vs. presidential) are included. Since winners-take-it-all arrangements produce a smaller number of winners (and thus a larger amount of losers) than consociational institutional arrangements, this also affects the balance of citizens that are satisfied with democracy.

#### Level of general corruption

In order to disentangle the effects of general corruption and specific ones, such as electoral fraud, we use the Transparency International’s *Corruption Perception Index* provided by *The Quality of Government* database (Teorell et al. 2012). We calculate a three-year average of this indicator in order to capture the influence of general corruption on respondents’ level of SWD.^4^

#### Other macro variables

Given the number of countries included in this study, we judge it prudent to control for factors that could account for variance in our dependent variable, such as level of economic development via GDP per capita (World Bank 2016).^5^ Socioeconomic development is a key control since it is often linked to changes in mass attitudes, including SWD (Diamond 1999). We also integrate items pertaining to economic policy performance, namely inflation rates measured through GDP deflators (World Bank 2016), as well as growth rates (World Bank 2016), both measured at the end of the year preceding the elections. Last, we use a measure capturing the average age of political parties from the *Database on Political Institutions* (Beck et al. 2001) to account for democratic maturation effects.^6^

## Analyses

### Aggregate models

**Figure 1.**
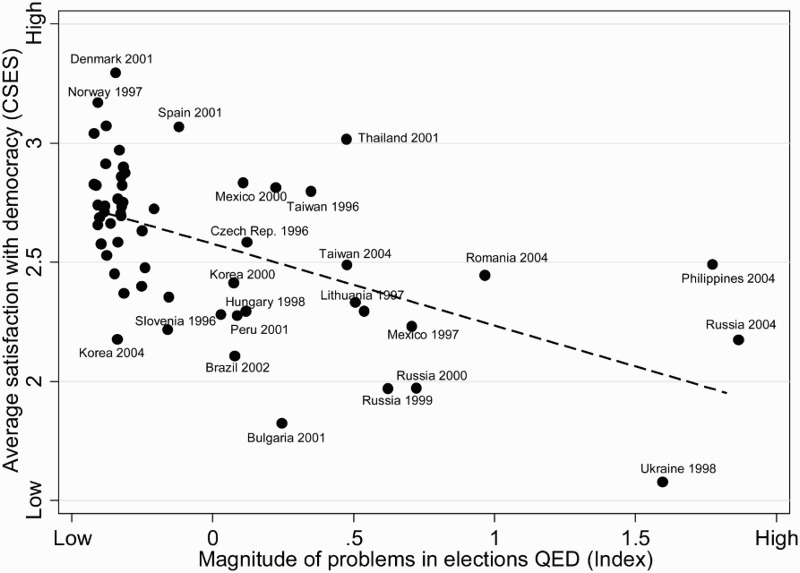
Overall magnitude of problems in elections in relation to average satisfaction with democracy. Sources: QED (Kelley 2010) and CSES. Figure contains 57 observations (elections). Pearson’s *r* = −0.55.

Figure 1 presents a bivariate relationship between observed levels of electoral fraud and citizens’ evaluations of democratic performance, measured as country averages. Upon first inspection, we notice a strong relationship between citizens’ average level of SWD and the overall magnitude of electoral fraud measured by our composite index. The majority of cases where heavy tempering with electoral proceedings took place is located in the bottom right quadrant of the graph and encompass states with lower degrees of SWD. Thinking of election fraud as a deficiency affecting the quality of institutions, our findings are consistent with analogous studies (e.g. Wagner, Schneider, and Halla 2008). However, the relationship illustrated in Figure 1 is not fully linear and presents some outliers, such as the 2001 parliamentary election held in Thailand. Even after violent protests emerged over vote counting, and allegations of vote-buying led to a rescheduling of the vote in 62 out of 400 constituencies, the average level of SWD in Thailand was, and remained, one of the highest in the group of countries considered.

Examination of Figure 1 also reveals that a large number of cases did not experience any problems in the conduct of elections for the period under study. Most of the countries in this group are established democracies that have a long history of holding free and fair elections. In those countries, the absence of electoral fraud is unlikely to significantly affect citizens’ SWD, as the size of the variance in SWD suggests. In the ensuing analyses, we therefore control for the status of emerging democracy by generating a dummy variable indicating whether a country is part of the third wave of democratization (following Huntington 1991).

Having established that electoral malpractice has an impact on SWD using aggregate measures, in the section below, we proceed with an examination of the effects of fraudulent activities on SWD using a cross-level research design to supplement the above findings.

### Multi-level models

The following analyses employ mixed-effects logistic regressions for ordered responses (random-effects intercept at the country level) to account for the hierarchical structure of the observations.^7^ All models are displayed in in Table 1. Model 1 is a baseline equation containing only individual-level predictors and our index of overall election quality. Models 2–5 incorporate the remaining country-level control variables. Electoral fraud has a negative impact on SWD, as highlighted by Model 1, although the coefficient does not achieve standard levels of statistical significance. This parameter estimate reaches statistical significance when controlling for the most important rival explanations in Models 2 and 3. In all models, we observe a statistically significant positive effect of winning an election on SWD, a finding that also bolsters the “winning” hypothesis. In addition, many of the control variables, for instance inflation and growth, are statistically significant in the hypothesized direction in most models, which lends confidence in the overall model specifications.

**Table 1.** Multi-level ordered logistic regressions modeling satisfaction with democracy.

	Model 1	Model 2	Model 3	Model 4	Model 5
	b/se	b/se	b/se	b/se	b/se
*Micro covariates*					
Age	0.998*** (0.00)	0.998*** (0.00)	0.998*** (0.00)	0.998*** (0.00)	0.998*** (0.00)
Gender	0.894*** (0.02)	0.896*** (0.02)	0.896*** (0.02)	0.896*** (0.02)	0.896*** (0.02)
Education	1.038*** (0.01)	1.040*** (0.01)	1.040*** (0.01)	1.040*** (0.01)	1.040*** (0.01)
Close to a political party (yes = 1)	0.956*** (0.00)	0.949*** (0.00)	0.948*** (0.00)	0.949*** (0.00)	0.949*** (0.00)
Left–right position	1.086*** (0.00)	1.082*** (0.00)	1.083*** (0.00)	1.083*** (0.00)	1.083*** (0.00)
Efficacy: who people vote for makes a difference	1.153*** (0.01)	1.151*** (0.01)	1.151*** (0.01)	1.152*** (0.01)	1.151*** (0.01)
Voted for winning party/coalition (yes = 1)	1.367*** (0.02)	1.331*** (0.02)	1.326*** (0.02)	1.308*** (0.03)	1.331*** (0.02)
*Macro covariates*					
Index of magnitude of problems in elections	0.952 (0.05)	0.547*** (0.03)	0.558*** (0.09)	0.588*** (0.04)	0.116*** (0.07)
GDP/capita		0.918** (0.03)	0.860** (0.12)	0.926** (0.03)	0.917** (0.03)
Lag*_t–1_* GDP deflator (inflation)		0.966*** (0.00)	0.965*** (0.01)	0.966*** (0.00)	0.967*** (0.00)
Lag*_t–1_* GDP growth		1.100*** (0.01)	1.091* (0.03)	1.104*** (0.01)	1.099*** (0.01)
Parliamentary (0), presidential (1)		0.586 (0.26)	0.777 (0.31)	0.587 (0.26)	0.787 (0.34)
Electoral rules (PR = 1)		0.657 (0.23)	0.696 (0.23)	0.658 (0.23)	0.707 (0.23)
Average age of parties		0.992*** (0.00)	0.996*** (0.00)	0.993*** (0.00)	0.992*** (0.00)
Third wave (yes = 1)		0.413** (0.14)	0.670 (0.21)	0.420** (0.15)	0.515** (0.17)
Corruption index (0 corrupt, 10 clean)			1.153*** (0.06)		
Winner × index of problems				0.895** (0.04)	
Third wave × index of problems					4.785** (3.09)
Observations	49,524	49,524	49,524	49,524	49,524
No. of election studies	48	48	48	48	48
No. of countries	29	29	29	29	29
Pseudo *R*^2^	0.041	0.074	0.077	0.075	0.075
Variance components	0.908 (0.242)	0.552 (0.150)	0.435 (0.118)	0.549 (0.149)	0.461 (0.124)
*BIC*	105,860.7	105,584.1	105,553.0	105,588.5	105,589.5

Note: Columns display exponentiated coefficients with standard errors (from exponentiated coefficients). Pseudo *R*^2^ estimated from conventional ordered logit.

*p < .10, **p < .05, ***p < .01.

**Figure 2.**
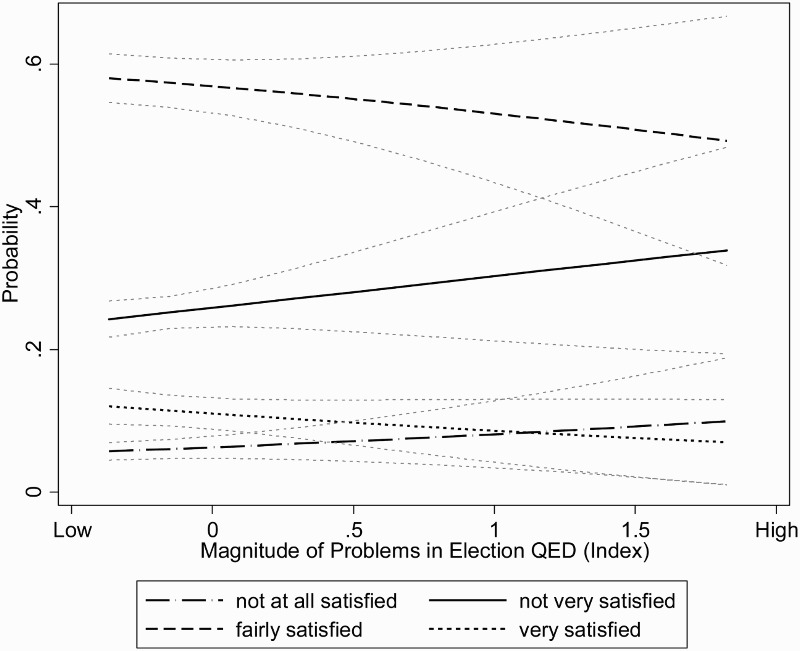
The effects of magnitude of problems in elections on satisfaction with democracy, predicted probabilities.

Note: Predicted probabilities estimated from model 3 in Table 1, holding all other variables at their means, 90% confidence intervals.

Looking at predicted probabilities, as illustrated in Figure 2, offers a clearer impression of the size of the effects of electoral fraud on SWD. The largest effects of electoral fraud are not registered in the extreme categories of the dependent variables: albeit in the hypothesized direction, the lines plotting predicted probabilities of being very satisfied or dissatisfied remain rather flat across different values of electoral fraud. Rather than in the extreme categories, electoral fraud exerts most of its impact in the middle categories – “fairly satisfied” and “not very satisfied.” Here the probability of not being very satisfied with democracy increases from about 0.25 to close to 0.35 as we move from the minimum to the maximum level of electoral fraud. As the level of problems observed in an election goes from “none” to “serious,” the probability of being fairly satisfied with democracy decreases from 0.58 to some 0.5. Both shifts represent substantial changes – close to 10 percentage points in predicted probabilities – over the values of electoral malpractice. Bearing in mind the modest overall explained variance of the models, the effect of electoral malpractice on SWD is considerable in the middle values of the dependent variable, which represents the tipping point between negative and positive feelings. Electoral fraud therefore has most impact on individuals holding lukewarm attitudes toward the way democracy works in their country, but does not seem to affect individuals located on the extremes. This is of particular relevance for countries undergoing processes of democratic consolidation, since success also hinges on the support of its citizens: those citizens who are “at the edge” of support or non-support are the critical group needed to support democratic consolidation, and the one most affected by electoral integrity.

We now turn to investigating the joint effect of electoral fraud and levels of corruption by adding this explanatory variable (see Model 3 in Table 1) to provide a conservative test as to whether electoral fraud can be empirically distinguished from conventional corruption. From Model 3, we see that the more a country is perceived to be free of corrupt practices (the indicator ranges between 0 – highly corrupt and 10 – clean), the likelihood for individuals to be in a high category of SWD also increases. Despite the addition of this variable, our measure of electoral fraud remains statistically significant. We interpret this result as an indication that voters are able to differentiate between corruption in general and electoral fraud in particular. While both of these phenomena negatively influence voters’ level of SWD, they remain empirically discernible from one another.

### Interaction effects

The coefficient for being on the winning side of the election retains a statistically significant impact on SWD throughout all model specifications. Overall, winning an election has a positive effect on SWD. Winning reduces the probability that respondents are not at all, or not very satisfied with democracy, and increases the probability that respondents are fairly, or very satisfied. To draw conclusions on the interplay of these two determinants of SWD, Model 4 introduces a multiplicative term composed of having voted for the winning party or not, and the index capturing the overall magnitude of problems in elections (cf. Table 1).

**Figure 3.**
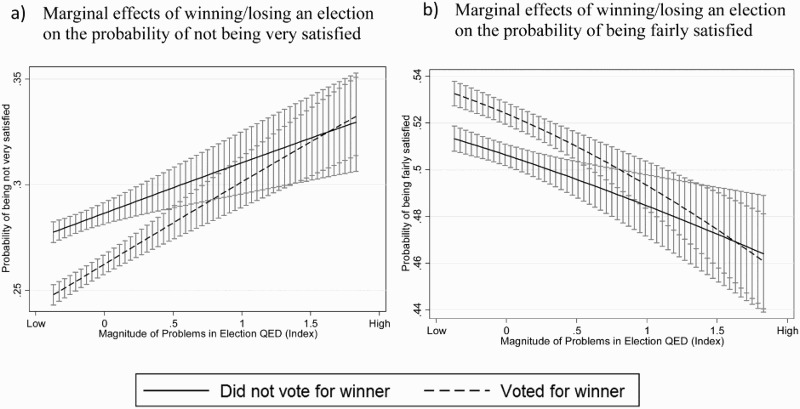
Marginal effects of winning and losing across magnitude of problems in elections on satisfaction with democracy.

Note: Marginal effects estimated from conventional ordered logit model including variables from model 4.

The interaction term between voting for the winning party and electoral malpractice is significant and negative, meaning that as elections become more problematic, the marginal effect of winning/losing decreases. The direction of the relationship goes against the body of literature arguing that winning has probably the highest possible utility and should therefore matter more than the degree of procedural fairness (Grimes 2006). To provide a substantive interpretation of these effects, we turn to graphical representations of marginal effects (Brambor, Clark, and Golder 2006). Figure 3 displays the marginal effects of winning an election on the probability of being more or less satisfied with the way democracy works at different levels of electoral fraud. Looking at the two figures representing the two middle categories of our outcome variable (being not very satisfied and being fairly satisfied), winning and losing do not display the same effects across the range of possible levels of electoral fraud. Having voted for the winning coalition rather than for a losing party only has noticeable effects on levels of SWD when elections are conducted in relatively impartial environment. Winners are more satisfied, and losers less satisfied with democracy, as long as elections are clean. As the overall quality of election declines and cross a certain threshold of malpractice, the lines for winning and losing intersect: winning and losing no longer matters. Our findings on the interplay between the level of electoral fraud and being on the winner’s side of an election therefore lead us to the conclusion that attitudes toward the functioning of democracy seem to stem from two sources dependent on the context of the elections. When elections are high in quality, expected policy gains dominate attitudes. However, as elections become marred with procedural problems, the working of democracy in the institutional sense – operationalized with the magnitude of problems in elections – becomes more important than winning or losing.

**Figure 4.**
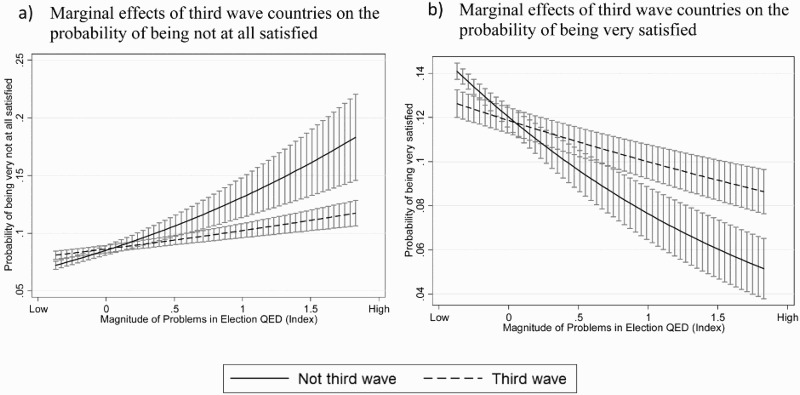
Marginal effects of third wave countries across magnitude of problems in elections on satisfaction with democracy.

Note: Marginal effects estimated from conventional ordered logit model including variables from model 5.

Our analyses also expose that electoral fraud does not affect voters in established versus newer democracies the same way. In Model 5, the addition of a multiplicative term between status as third wave-democracy and the index of overall election quality reveals some interesting patterns, as illustrated in Figure 4.^8^ The levels of SWD are higher in third wave democracies than in other countries, irrespective of the magnitude of problems in elections. The marginal effects graph of this variable (Figure 4(a)) demonstrates that as electoral quality declines, the increase in likelihood of being “not at all satisfied” is steeper in established democracies than in the newer democracies. The reverse is also true (Figure 4(b)): the likelihood of being very satisfied with democracy decreases more sharply as the quality of elections decreases for the group of older democracies than for the third wave democracies. Despite experiencing higher levels of electoral malpractice, our results show that, paradoxically, voters’ levels of SWD in third wave democracies are less sensitive to electoral malpractice than those in the first and second waves.

## Conclusions

In this article, we sought to examine the effects of electoral fraud on voters’ attitudes toward the functioning of democracy in their country, using a broad comparative research design based on two of the CSES Modules supplemented with contextual data. Our research design allowed large enough statistical power to test our hypotheses using both macro-level and micro-level observations. Our analyses offer additional – and more direct – empirical support for the hypothesis that electoral misconduct has a considerable effect on attitudes toward the functioning of democracy which to date had largely relied on voters’ perceptions of electoral fraud rather than objective measurements. The combined observation of problems in the legal framework, political and administrative problems in the pre-election period, and the integrity of elections during election day produce a negative effect on levels of SWD. This effect remains stable even when controlling for other potentially influential variables, such as economic circumstances, different institutional contexts, and general corruption levels. Electoral fraud is therefore likely to shape perceptions of accountability and responsiveness, which are key factors explaining regime support (Aarts and Thomassen 2008; Anderson and Guillory 1997; Klingemann 1999; Mischler and Rose 2001).

Our findings also side with the body of scholarship arguing that impartial bureaucratic procedures are more consequential for SWD than electoral outcomes in terms of winning and losing (Rothstein 2009; Rothstein and Teorell 2008). The relationship between voting for the winning party, coalition, or candidate and SWD, observed by so many contributors, only holds in the context of free and fair elections. We find that as electoral malpractice increases, winners and losers no longer display different levels of SWD. Electoral malpractice affects the perceptions of citizens in the same direction, no matter if they are on the winning or losing sides.

If satisfaction is rationally based and hinges on citizens’ evaluation of the performance of governments, the cost of electoral malpractice is high since it negatively impacts performance evaluations of governments, and ultimately could affect regime stability in emerging democracies. We find that electoral fraud does not affect voters the same way in established versus newer democracies: newer democracies are more likely to experience electoral malpractice, yet levels of SWD in these countries are less sensitive to the quality of elections than those in more established democracies. Nonetheless, since broad support for democratic values is an underlying condition for the consolidation of democracy (Almond and Verba 1963; Linz and Stepan 1996; Lipset 1994; Norris 1999), widespread electoral fraud – considering its effects on attitudes – could result in particularly inauspicious climates for the survival of new democracies. This finding raises a number of questions as to why electoral malpractice would be perceived more benignly in new democracies, and about the perception of procedural fairness as a continuous process over time rather that at single time point.

Regardless of these interesting and in parts paradoxical findings, we acknowledge that the data available to measure electoral fraud are limited in qualitative and quantitative terms. Yet our study paves the way for new research agendas on the effects of electoral fraud on citizens. As data become more readily available and reliable, we think an extension of our analyses to include cross-temporal comparisons would be the next logical step. Last, the link between “objective” evaluations of electoral fraud and the public’s perceptions of electoral integrity, as well as performance evaluations should be further scrutinized. Given the importance of perceived electoral fairness on citizens’ evaluation on the way democracy works in their countries, the perception of political legitimacy, and turnout, it remains unclear when, or at which point, objective malpractice is translated into subjective evaluations.

## Footnotes

1 This number of elections and countries results from combining various data sources: QED data, the CSES, and other aggregate data sources (Corruption Perception Index, World Bank, Dataset on Political Institutions). The countries and years included are located in Appendix 1.

2 The composite index we use was constructed by standardizing and summing the following items from QED: freedom to change government, freedom of association, freedom of speech, overall election quality, extent of problems, legal environment, pre-election political conditions, pre-election administrative capacity, pre-election violence, polling day cheating, polling day violence, and polling day administrative capacity.

3 As a robustness check, we have performed all analyses included in this article using two alternative measurements of electoral fraud to insure our results are not data source dependent. These models are located in Appendix 2, Tables A2 and A3, and reveal that data choice has a modest impact on the findings of this paper.

4 For Lithuania (1997), Romania (1996), and Slovenia (1996), a single subsequent time point was retained, since these countries are not covered by any data source in these particular years.

5 A certain level of multicollinearity is expected between the macro parameter estimates as several of our control variables are related to each other. We regard this as unproblematic as the variance inflation factor for our main variable of interest, electoral fraud, remains below 2.5 in all models.

6 We have also tested for the effects of an additional control variable, the margin of victory of the winning party or candidate using two different specifications, in order to ascertain the effect of election problems on SWD is not spurious. Given that the addition of this variable (presented in Appendix 3, Tables A4–A5) positively affects the strength of our findings, rather than absorbing their explanatory power, we chose to follow a conservative research strategy in omitting this variable.

7 Given the few repeated country observations (48 election studies nested in 29 countries), most country-level factors would be absorbed by the use of country-level fixed effects for non-repeated cases, including our main independent variable depicting the quality of elections. To verify that our modelling choice does not impact results, we present alternative specifications using fixed effects models with country dummies in Appendix 4, Table A6. Despite the massive collinearity introduced by the country dummies, our main results hold.

8 We wish to note, however, that the significance of this coefficient is sensitive to data source on electoral fraud.
